# Supplementary material for: Prognostic role and functional impact of cadherin genes in non-small cell lung cancer tumorigenesis: mechanistic insights from in silico and in vitro analyses
Source: PeerJ. 2025 Aug 19;13:e19785. doi: 10.7717/peerj.19785 (PMC12372785; doi:10.7717/peerj.19785)
Supplement: Supplemental Information 5 [file peerj-13-19785-s005.docx]

# Suppl data Table 1: Mean ΔCT values from RT-qPCR CCK8 assay

| Condition | Mean ΔCT Value |
| --- | --- |
| Ctrl-CDH1-A549 | 8.7 |
| Ctrl-CDH2-A549 | 11.8 |
| si-CDH1-A549 | 5.0 |
| si-CDH2-A549 | 6.0 |

# Suppl data Table 1: Mean OD values from CCK8 assay

| Time (h) | Ctrl-A549 (OD) | si-CDH1-A549 (OD) | si-CDH2-A549 (OD) |
| --- | --- | --- | --- |
| 24 | 0.700 | 0.406 | 0.364 |
| 48 | 1.000 | 0.580 | 0.520 |
| 72 | 1.300 | 0.754 | 0.676 |


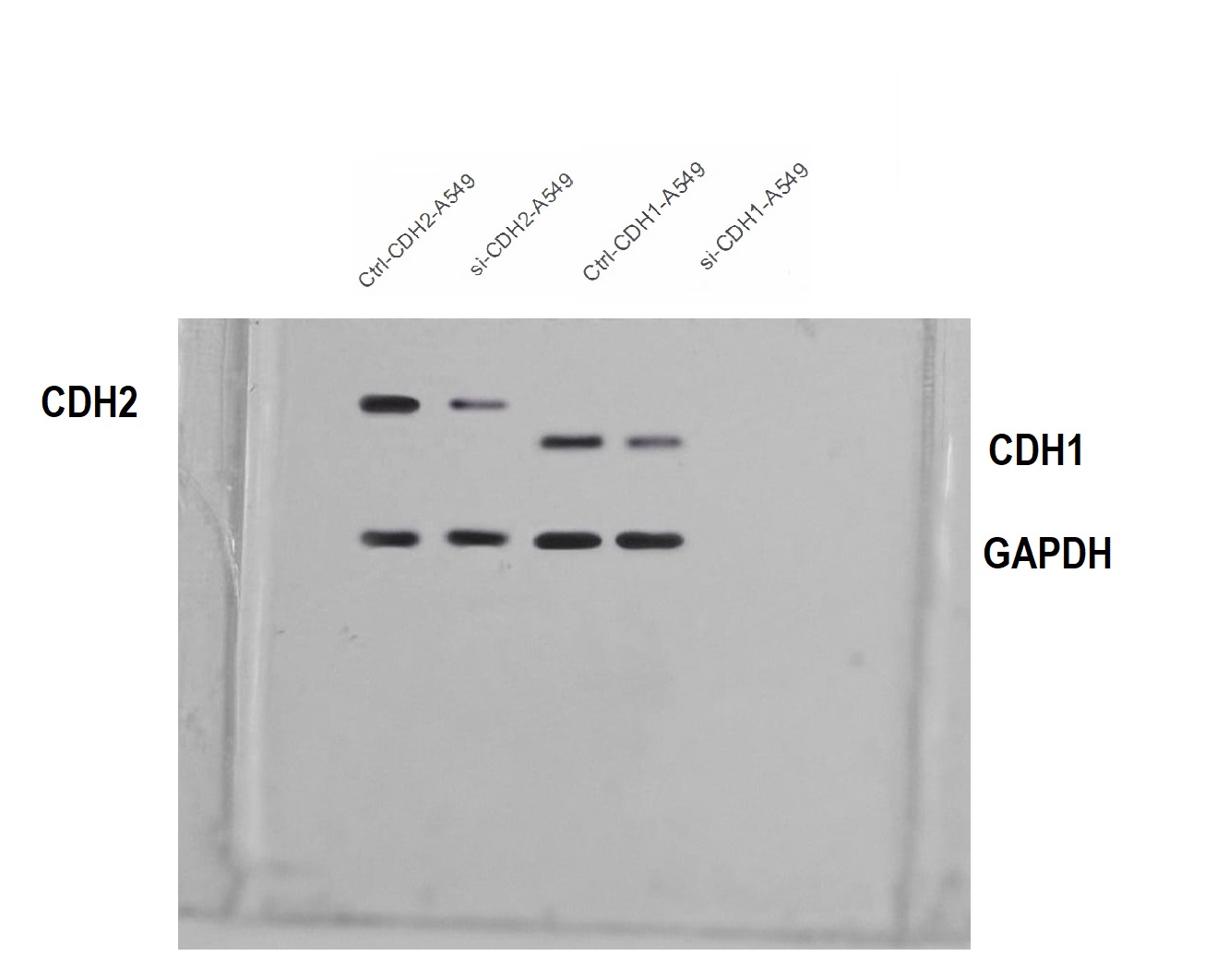


**Suppl data Figure 1: Uncut Western blot bands of GAPDH, CDH1, and CDH2 across A549 cells.**
